# Compliance with transmission-based precautions, and associated factors among healthcare providers in Cameroon: a cross-sectional study

**DOI:** 10.1186/s13756-025-01523-8

**Published:** 2025-03-11

**Authors:** Leslie Tasha Mbapah, Midrelle Syntyche Tsague, Denise Georges Teuwafeu, Mbapah Tracy Ngwanui, Sandra Tabe Etaka, Fombo Enjeh Jabbossung, Brandon Carl Monika Pouekoua, Longsti Scarlet Tabot Enanga, Taljaard Jantjie

**Affiliations:** 1 Triad Research Foundation (TRF), Buea, Cameroon; 2 Faculty of Health Sciences, University of Buea, Buea, Cameroon; 3 Université Libre de Bruxelles, Brussels, Belgium; 4 Liverpool School of Tropical Medicine, Liverpool, UK; 5 Faculty of Medicine and Health Sciences, Stellenbosch University, Cape Town, South Africa

**Keywords:** Healthcare-associated infection, Healthcare providers, Infection prevention and control, Antimicrobial resistance, Personal protective equipment, Compliance, Cameroon

## Abstract

**Background:**

Transmission-based precautions (TBP) and the proper use of personal protective equipment (PPE) are essential in preventing hospital-acquired infections (HAIs) and in controlling the emergence and spread of antimicrobial resistance (AMR). This study, therefore, aimed to determine healthcare providers’ compliance with TBP and its determinants in healthcare settings to help curb the burden of HAIs and AMR.

**Method:**

This study was a cross-sectional, hospital-based research conducted among healthcare providers at four health facilities in the Fako division of Cameroon, from January 1 to May 31, 2024. A standardized observation form, adapted from the World Health Organization’s checklist for hand hygiene practices, was used to assess compliance with Transmission-Based Precautions (TBP) among healthcare providers when interacting with patients known or suspected of having infectious pathogens. Multivariable logistic regression analysis was performed to identify factors independently associated with TBP compliance, with significance set at a p-value of less than 0.05.

**Results:**

The proportion of participants with good TBP compliance was **75.4%** (95%CI: 67.4–82.2). Contact precaution compliance was 94.2%, while that for droplet /airborne was 12.8%. Factors independently associated with good TBP compliance were healthcare providers trained in IPC **(aOR: 2.89, 95%CI: 1.16—7.22),** the availability of PPE in the facility’s departments **(aOR: 6.00, 95%CI: 1.24–29.17),** and working in the facility; Mount Mary Hospital **(aOR: 22.47, 95%CI: 2.21–228.08).**

**Conclusion:**

Compliance with transmission-based precautions was suboptimal. The determinants of good compliance with TBP among healthcare providers were making PPE available in the facility and training healthcare providers on IPC. Tailored public health measures should be implemented to improve and sustain healthcare providers’ compliance with TBP.

**Supplementary Information:**

The online version contains supplementary material available at 10.1186/s13756-025-01523-8.

## Background

The healthcare facility environment is structured to deliver safe and effective healthcare. Still, it is also a well-recognized reservoir of infection responsible for many healthcare-associated infections (HAI) and antimicrobial resistance (AMR) affecting both patients and healthcare providers (HCPs) [1]. HAI is contracted in the hospital or other healthcare settings and manifests at least 48 h after hospitalization and should not be incubating at the time of admission [2]. This contributes significantly to morbidity and mortality. Bacteria associated with HAI are mostly antimicrobial-resistant [3]. According to the World Health Organization (WHO), the spread of multi-drug resistant microbes in healthcare settings is frequent and mostly occurs through HCPs’ contaminated hands or equipment and environment, which leads to outbreaks [4]. Robust infection prevention and control (IPC) measures, which include the use of personal protective equipment (PPE), are effective in controlling the spread of AMR, and it’s one of the objectives of the WHO’s Global Action Plan on AMR [5, 6].

The pooled global prevalence of HAI between 2000 and 2021 was 0.14%, with an annual rate increase of 0.06%. The Central African region had the highest rate of HAI, estimated at 0.27% [7]. Systematic review and meta-analyses in 2022 and 2024 revealed the prevalence of HAI in Africa at 12.76% and 15%, respectively [3, 8].

IPC measures aim to prevent infection in the healthcare environment and reduce infection transmission [9]. Standard precaution (SP) and transmission-based precaution (TBP) are the two tiers of IPC measures for protecting patients and HCPs against HAI. These measures include hand hygiene practices and the use of specialized material known as PPE, amongst others [10, 11]. Some studies have, however, suggested poor compliance with the use of PPE [12]. SP is recommended in all patients to avoid contact with bodily fluids regardless of suspected or confirmed status [13]. At the same time, TBP is used in addition to SP for infection prevention in patients who may be colonized or infected with specific infectious agents, which warrants additional infection transmission prevention via airborne, droplet, or direct/indirect contact [14].

Many tools have been developed to assess healthcare workers’ compliance with IPC measures. However, this has mainly been done for SP as opposed to the very few on TBP. These tools widely used are self-reported with varied methodologies and tend to overestimate compliance [15, 16]. Moreover, it was reported by Lommi et al. that these available instruments that measure the compliance of HCPs with SPs are of low-moderate quality [16]. A more objective measure of HCPs’ compliance to SP, developed by the WHO for direct observation of HCPs during patient care by trained and validated observers, is considered the gold standard for hand hygiene monitoring [17].

A study in Cameroon revealed challenges to adhering to IPC measures by HCPs, notably high workload, distant washing points, the lack or erratic availability of PPE, as well as the perceived risk-free nature of care activity [18]. Another study in Cameroon evaluating compliance to facility-level attributes like awareness and adoption of the national IPC guidelines, availability of isolated rooms, composition/functioning of IPC committees, staff training on IPC, and IPC surveillance activities reported less than 50% compliance with all facilities [19].

Despite these challenges, there is a dearth of data concerning HCPs’ compliance with TBP in Cameroon and Africa. Addressing this gap will help drive policy changes to curb the spread of HAI and AMR.

## Methods

### Aim

This study aimed to determine healthcare providers’ compliance with TBPs (appropriate use of PPE) and their determinants in four health facilities to help curb the burden of HAIs and AMR.

### Study design

This was a cross-sectional study conducted with HCPs over five months (1st January to May 2024) in four health facilities in the Fako division of the Southwest region of Cameroon.

### Study setting

Health facilities in Cameroon are categorized into seven categories. The first category is general hospitals, the second category is central hospitals, the third category is regional hospitals, the fourth category is district hospitals, the fifth category is subdivision medical centers, the sixth category is integrated health centers, and the seventh category is ambulatory health centers. A national IPC guideline is made available to all health facilities nationwide by the Ministry of Health [20]. Convenient sampling was used to select the facilities, considering their high bed capacities and whether they are publicly or privately funded. The publicly funded were Buea Reginal Hospital (Category three, which has a sanitation department but without statutory meetings and follow-ups) and Limbe Regional Hospital (Category three, which has an IPC committee with neither a specified meeting period nor regular follow-up), and two private hospitals, Mount Mary Hospital(Category four, which has an IPC committee with regular monthly meetings) and Solidarity Health Foundation (Category four, which has makeshift IPC committee when need arises).

### Study population

HCPs who had contact with patients or potentially infectious samples in the four facilities, had worked for at least six months, and consented to the study were recruited. HCPs were randomly chosen from each facility through proportionate-to-size sampling. 

The sample size calculation for a single proportion (n = z^2^pq/e^2^) was used. Where z = 1.96, e = level of precision at 5%, and p = 0.9 is the full compliance for SP at 90% reported in a survey in 2016 by Hassan Haridi et al., in Saudi Arabia [21]. The calculated minimum sample size was 139 participants.

### Proportionate sampling of participants from the facilities

Proportionate sample per facility; **nf/N**, where n = minimum sample, f = HCPs in the departements considered per facility, N = Total HCPs in all four facilities. Buea Regional Hospital minimum sample population: $\frac{{139 \times 195}}{{607}} = 44.65\approx45$.

Limbe Regional Hospital minimum sample population: $\frac{139\times 225}{607} = 51.52\approx52$.

Mount Mary Hospital minimum sample population: $\frac{139\times 130}{607} = 29.77\approx30$.

Solidarity Health Foundation minimum sample population: $\frac{139\times 65}{607} = 14.88\approx15$. Therefore, 142 participants who had indications for TBPs during the individual observation period were recruited. 

### Data collection

This study used a structured questionnaire that captured sociodemographic characteristics and knowledge level on IPC measures (10-item questions on IPC measures) [22–24]. The compliance with TBP was obtained by direct HCP observation while they worked on patients with known transmissible diseases (TB, HIV, pneumonia, Hepatitis B and C, suspected measles, and suspected cholera). The compliance was obtained using an observation checklist adapted from the WHO hand hygiene observation form [17] (see Additional file 1) adapted to capture measures tailored to TBPs. (see additional file 2).

To validate the questionnaire and the adapted WHO observation forms, two researchers pretested them in a different facility among 15 HCPs, and corrections were made to capture TBP measures accurately. The HCPs were invited and given the study information sheets to help them understand the study, after which they signed the consent forms. Data collection started with participant observations for an average of 20 min each during healthcare delivery, with three opportunities for TBP noted per participant. For each opportunity, an indication and whether an action was taken or not was noted, which involved the use of gloves, gown, face mask, goggles, aprons, etc., depending on the specific TBP indication in a defined field of observation (patient immediate vicinity of care whose dimensions varied with the facility, e.g., a ward, room) of the observer determined before the initiation of the observation. The observation was performed only once, and this was done for each selected HCP in the chosen department, covering all work shifts. It was considered that no TBP action was taken if the HCP left the observer’s field of observation without performing an indicated action.

The self-administered structure questionnaire was conducted immediately after the participant observation.

This structured questionnaire captured data on sociodemographic characteristics, IPC-related information of the participants, department, and facility, and lastly, a 10-item questionnaire to assess their knowledge level on IPC [25] (see Additional file 3).

### Description of variables

#### Outcome variable

Compliance with TBPs.

Calculated as; Compliance = (number of Actions)/(Total number of Opportunities) × 100. This was done per HCP per session, which lasted an average of 20 min.

A cut-off for good compliance with TBP was set at an overall score of ≥ 80%, in line with the threshold used for SP compliance by Bahegwa et, 2022 in Tanzania [26]. None was found in the literature for TBP.

#### Explanatory variables

*Participant factors*: Gender (male, female), Age in years, work status (contract, volunteer, state worker), trained in IPC (Yes, No), profession (Doctor, Nurse, Midwife, Laboratory Technician), knowledge level on IPC (knowledgeable, not knowledgeable). Being knowledgeable was set at a score ≥ 7/10 since the mean knowledge score in our study was 7.25.

*Facility factors*: PPE available (Yes, No), IPC guideline in the department (Yes, No), IPC committee in the facility (Yes, No), facility type (public-funded, private-funded), facility (Buea Regional Hospital, Limbe Regional Hospital, Solidarity Health Foundation, Mount Mary Hospital), Department [Surgical, Medical, paediatric, Obstetrics and gynaecology (OBGYN), Outpatient, Private ward, Laboratory].

### Data analysis

The data was entered into Excel 2016 for cleaning. The cleaned data was then exported into StataMP 18.0 for analysis. Categorical variables were computed and presented as proportions and percentages, and quantitative variables as means with standard deviation (SD). The Chi-square test was used to compare proportions. Multivariable logistic regression analysis with backward elimination (likelihood ratio p values) was used to identify factors independently associated with good compliance with TBP. Covariates with *p* ≤ 0.20 were fitted into the model. The covariate professional cadre was not included in the model due to collinearity with department. Multicollinearity was checked with the mean–variance inflation factor (VIF) of 1.61, and the model fitness was tested with Hosmer–Lemeshow statistics (*p* = 0.40). The adjusted odd ratios and 95% confidence intervals were reported with their *p*-values. The level of significance was set at *p*-value < 0.05.

## Results

Of the 142 HCPs included in the analysis, the majority were females, 106 (74.5%), and nurses, 75 (52.8%). The mean age of the participants was 27.35 years (SD ± 6.73). Most of the HCPs were from publicly funded health facilities, 101 (71.1%) and 131 (92.3%) indicated the availability of PPE in their departments. Also, 108 (76.6%) reported the presence of an IPC committee in their facilities, and 130 (91.5%) reported the availability of IPC guidelines in their departments. About 3 out of 4 participants had training in IPC, and 45.1% were knowledgeable on IPC. See Table 1.

**Table 1:** General characteristics of study participants in four health facilities in Fako, Cameroon

Variable	Frequency (n = 142)	Percentage (%)
*Participant factor*		
Gender		
Male	36	25.4
Female	106	74.6
Age (in years)	27.35 ± 6.73	
18 to ≤ 25	73	51.4
> 25 to ≤ 35	54	38.0
> 35	15	10.6
Profession		
Doctor	22	15.5
Lab. Technician	32	22.5
Midwife	13	9.2
Nurse	75	52.8
Work status		
Volunteer	89	62.7
Contract	39	27.5
State worker	14	9.8
Training in IPC		
Yes	107	75.4
No	35	24.6
Knowledge of IPC		
Knowledgeable	64	45.1
Not knowledgeable	75	54.9
Covid-19 vaccination		
Yes	46	32.4
No	96	67.6
Hepatitis B vaccination		
Yes	101	71.1
No	41	28.9
*Health Facility factor*		
Facility		
Buea Regional Hospital	45	31.7
Limbe Regional Hospital	52	36.6
Mount Mary Hospital	30	21.1
Solidarity Hospital	15	10.6
Type of facility		
Public	101	71.1
Private	41	28.9
Department		
Surgical unit	37	26.1
Laboratory	33	23.2
Medical	21	14.8
OBGYN	21	14.8
Outpatient	16	11.3
Pediatric	09	6.3
Private ward	05	3.5
PPE present unit		
Yes	131	92.3
No	11	7.7
IPC committee present		
Yes	108	76.6
No	34	23.9
IPC guidelines present in the unit		
Yes	130	91.5
No	12	8.5

OBGYN; Obstetrics and gynaecology, PPE; Personal protective equipment; IPC; Infection prevention and control, % Percentage, n; Sample

In this study, the proportion of participants with good TBP compliance was **75.4%** (95%CI: 67.4–82.2).

The predominant TBP type was contact precaution 125 (88.0%), followed by droplet/airborne 17 (12.0%). The compliance to contact precaution was 94.2%, while for droplet /airborne was 12.8% (*P* = 0.09).

### Proportion of compliance with TBP in facilities, departments, and professional cadre

**Fig. 1:**
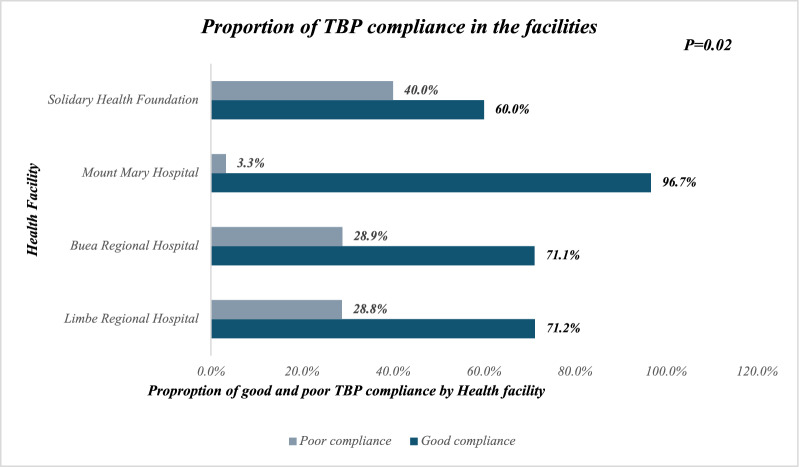
Proportion of transmission-based precautions (TBP) compliance among healthcare providers in the four health facilities

The highest proportion of HCPs with good compliance with TBP were from Mount Mary Hospital (96.7%), and close to three-quarters of participants from Limbe and Buea Regional Hospitals had good compliance with TBP (***P* = 0.02**). See Fig. 1.

**Fig. 2:**
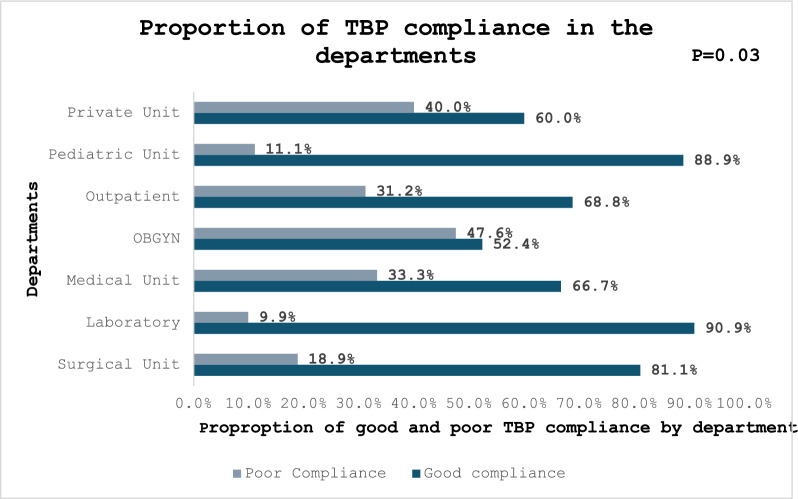
Proportion of transmission-based precautions (TBP) compliance among healthcare providers in the facility departments

The department with the highest proportion of HCPs with good compliance with TBP was the laboratory (90.9%) and the pediatric unit (88.9%), followed by the surgical unit (81.1%). Only 1 in 2 obstetrics and gynecology unit (OBGYN) participants had good compliance with TBP (***P* = 0.03**). See Fig. 2.

**Fig. 3:**
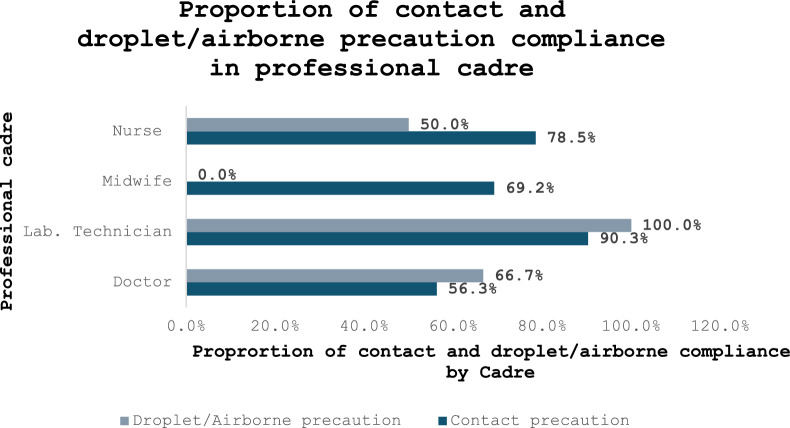
Proportion of contact and droplet/airborne precautions compliance in each professional cadre in the four health facilities

The professional category with the highest proportion of TBP compliance was Laboratory technicians (90.6%), followed by Nurses (75.7%), and the least were Doctors (59.1%) (*P* = 0.06). For contact precautions, Laboratory technicians have the highest compliance (90.3%), followed by Nurses (78.5%). Meanwhile, for droplet/airborne precautions, Laboratory technicians (100.0%) also had the highest compliance percentage, followed by Doctors (66.7%). See Fig. 3.

### Indications for transmission-based precautions

The most common reason for needing TBP in our study was blood draw (31.7%), closely followed by wound dressing (30.3%), both of which warranted contact precautions. Meanwhile, cough, which needed droplet/airborne precaution, was 12.0% (see Table 2).

**Table 2:** Indications for transmission-based precautions among healthcare providers in four health facilities in Fako Cameroon

Indication	Frequency (n = 142)	Percentage (%)
Blood draw	45	31.7
Wound dressing	43	30.3
Cough	17	12.0
Childbirth	14	9.9
Vomiting	08	5.6
IV-line placement	06	4.2
Bleeding	05	3.5
Watery stool	04	2.8

IV; Intravenous, %; Percentage, n; Sample

### Factors associated with good compliance with transmission-based precautions

On univariable analysis, work status, availability of PPE in the departments, facility, type of facility, department, availability of IPC guidelines in the departments, IPC training, and IPC committee in the facility were eligible for the multivariable model.

On multivariable analysis, factors independently associated with good TBP compliance were HCPs trained in IPC **(aOR: 2.89, 95%CI; 1.16–7.22, *P* = 0.02),** the availability of PPE in the facility’s departments **(aOR: 6.00, 95%CI; 1.24–29.17, *P* = 0.03),** and working in the facility; Mount Mary Hospital **(aOR: 22.47, 95%CI; 2.21–228.08, *P* = 0.008).** See Table 3.

**Table 3:** Factors associated with TBP compliance among healthcare providers in four health facilities in Fako, Cameroon

Variables	Univariable analysis (n = 142)			*P* value	Multivariable analysis (n = 142)			*P *value
	%	OR	(95%CI)		%	aOR	(95%CI)	
Gender								
Male	74.6	1.49	(0.59–3.77)	0.40	–	–	–	–
Female	25.4	1						
Age (in years)								
18 to ≤ 25	51.4	0.38	(0.04–3.19)	0.37	–	–	–	–
> 25 to ≤ 35	38.0	0.75	(0.07–7.88)	0.81	–	–	–	–
> 35	10.6	1						
Work status								
Contract	27.5	3.5	(0.74–16.55)	0.11	27.5	3.25	(0.46–22.76)	0.24
Volunteer	62.7	0.92	(0.26–3.19)	0.89	9.8	0.97	(0.19–4.89)	0.97
State worker	9.8	1			62.7	1		
PPE present in unit								
Yes	92.3	4.22	(1.20–14.83)	**0.03**	92.3	6.00	(1.24–29.17)	**0.03**
No	7.7	1			7.7	1		
Facility								
Buea Regional Hospital	31.7	1.64	(0.49–5.55)	0.43	31.7	1.34	(0.38–4.76)	0.65
Limbe Regional Hospital	36.6	1.64	(0.50–5.43)	0.41	36.6	2.20	(0.61–7.92)	0.23
Mount Mary Hospital	21.1	19.33	(2.05–182.55)	**0.01**	21.1	22.47	(2.21–228.08)	**0.008**
Solidarity Hospital	10.6	1			10.6	1		
Type of facility								
Public	71.1	0.43	(0.16–1.12)	0.08	71.1	0.45	(0.09–4.19)	0.49
Private	28.9	1			28.9	1		
Department								
Surgical Unit	26.1	2.86	(0.40–20.47)	0.30	26.1	3.33	(0.42–26.45)	0.26
Laboratory	23.2	6.67	(0.78–57.06)	0.08	23.2	3.86	(0.42–35.19)	0.23
Medical unit	14.8	1.33	(0.18–9.91)	0.78	14.8	1.27	(0.15–10.75)	0.82
OBGYN	14.8	0.73	(0.10–5.33)	0.76	14.8	0.74	(0.09–6.11)	0.78
Outpatient unit	11.3	1.47	(0.18–11.72)	0.72	11.3	0.92	(0.10–8.61)	0.94
Pediatric Unit	6.3	5.33	(0.34–82.8)	0.23	6.3	10.89	(0.57–209.16)	0.11
Private Ward	3.5	1			3.5	1		
Knowledge on IPC								
Knowledgeable	45.1	0.97	(0.45–2.08)	0.93	–	–	–	–
Not knowledgeable	54.9	1						
IPC Guideline present in unit								
Yes	91.5	3.48	(1.04–11.62)	**0.04**	91.6	1.30	(0.14–12.14)	0.82
No	8.5	1			8.4	1		
IPC Committee present								
vYes	76.6	2.02	(0.87–4.67)	0.10	76.6	1.45	(0.36–5.81)	0.60
No	23.9	1			23.9	1		
Training in IPC								
Yes	75.4	3.26	(1.43–7.46)	**0.005**	75.4	2.89	(1.16–7.22)	**0.02**
No	24.6	1			24.6	1		

PPE; Personal protective equipment, IPC; Infection prevention and control, n; Sample, OR; Odds ratio, aOR; Adjusted odds ratio, %; Percentage, CI; Confidence interval

## Discussion

This study set out to determine the compliance of HCPs with TBPs and the associated factors. Three-quarters of the HCPs had good compliance with TBP. Factors independently associated with compliance with TBP were training in IPC, PPE availability, and working in Mount Mary Hospital.

Compliance with TBP was 75.4% in this study. This is suboptimal since it’s below the ≥ 80% for good IPC compliance, according to Bahegwa et al., 2022 in Tanzania [26]. This could be because we used a direct observation method in this study, whereas they used compliance to standard precautions scale (CSPS), a self-reported questionnaire that potentially overestimates compliance. This finding is similar to Kim and Lee’s observed adherence rate of 76.8% reported in 2022 among HCPs in South Korea [27]. However, it contrasts with the high (90.6–97.5%) adherence to appropriate PPE reported by Ashinyo et al. in Ghana [28]. This is because this study considered compliance as the use of PPE when TBP is indicated in the post-COVID-19 era. In contrast, they determined compliance with PPE during the COVID-19 era, where HCPs were more conscious of the risk of transmission of the highly infectious SAR-COV 2 virus. It is worth noting that this is higher than the IPC compliance of 50.7% by Angaw et al. in [26] and 34.5% by Senbato et al. in 2014 in Ethiopia [24]. The higher compliance in our study can be explained by the fact that we studied compliance with TBP, whereas they reported compliance with SP. This difference could be because HCPs in our study may have been more conscious when working with patients with known or suspected to be infected with a transmissible disease.

PPE was available for most of the participants in this study. This is in contrast to less than half of the participants reporting the availability of PPE in a study in the capital city of Cameroon [18]. This is possible because the majority of the participants in our study were from secondary referral hospitals. In contrast, their study participants were recruited from district hospitals, which may be less equipped. In this study, the availability of PPE was significantly associated with good compliance with TBP. This is congruent with the findings of Angaw et al. in 2019 in Ethiopia, where a sufficient supply of protective material was associated with adherence to IPC [29]. The safety culture of making PPE readily available to HCPs enables usage when indicated and will aid in curbing HAI and, hence, AMR emergence and spread.

Compliance with contact precautions in this study was high (94.2%). This may be because infectious bodily fluids from patients, such as diarrheal stool, blood, and wound discharge, were visible to the HCPs, who took extra precautions to protect themselves, unlike the 12.8% compliance for droplet/airborne precaution. This is in contrast with the low compliance (7–22%) with contact precaution reported by Yanke et al. in the USA [30]. This difference can be explained by the fact that this study considered appropriate use of PPE when indicated, whereas they took into account full compliance with room entry and exit, donning /doffing of PPE, and elements of SP.

In this study, Doctors’ compliance with contact precautions was low (56.3%). This is in contrast to the high infectious disease doctors’ contact precaution compliance of 100% reported by Katanami et al., 2018 in Japan [31]. This is because, in this study, the Doctors were all non-infectious disease specialists who are not experts in infectious disease control and management.

Most of the staff working in the laboratory had good compliance with TBP in our study. This is the same as the finding reported by Gebresilassie et al. in 2014 in Northern Ethiopia [13] where laboratory staff had higher odds of compliance with IPC measures. However, department and professional cadre were not significantly associated with TBP which is similar to that reported by Mutaru et al. in 2022 in Ghana [25] where professional rank was not associated with IPC compliance.

In our study, HCPs from Mount Mary Hospital, a private facility, had higher odds of good compliance with TBP. This is similar to the finding of Tyagi et al. in India [32] where they found that private facilities had 100% compliance with IPC (hand hygiene), compared to public facilities, which had 27%. This could be because Mount Mary Hospital is the only facility in our study with a functional IPC committee that meets monthly. In addition, the administration and staff are possibly more conscious about the quality of care as they are privately funded and rely on patient turnout for sustainability.

Being knowledgeable about IPC was not significantly associated with TBP. This is similar to the findings by Mutaru et al. in Ghana in 2022 [25]. This is because knowledge does not directly translate into practice, as other factors like attitude, organizational safety culture, and perceived benefit have been shown to influence IPC practice [33, 34]. However, this was in contrast with the report by Senbato et al.in Ethiopia [24] and a systematic review and meta-analysis by Alhumaid et al. in [35] where knowledgeable participants had higher odds of IPC compliance. Differences in tools to measure knowledge, grading, and study methodology could explain the difference observed.

The availability of IPC guidelines (91.5%) was associated with TBP on univariable analysis, but this disappeared when controlled for other covariates in our study. This contrasts with the findings by Mutaru et al. in Ghana [25] where the presence of IPC guidelines in the department was associated with IPC compliance. The availability of IPC guidelines is expected to improve IPC compliance since it is a source of continuous knowledge and reminders.

HCPs who had training in IPC had higher odds of compliance with TBP. This is in agreement with the results of Senbato et al. 2024 in Ethiopia and Bahegwa et al. 2022 in Tanzania [26]. Training HCPs in up-to-date IPC practices is necessary to prevent and control HAIs [11].

### Study strength and limitation

This study utilized a direct observation method to obtain compliance, which is considered the gold standard by WHO, over a self-reporting that overestimated compliance to IPC.

Our study was subjected to the Hawthorne effect, as HCPs could change their practice (social desirability) if they were aware of being observed. However, we tried to mitigate this by allowing at least 24 h to elapse after obtaining the informed consent and only administering the questionnaire after the direct observation.

## Conclusion and recommendations

In our study, three-quarters of HCPs had good compliance with TBPs, which was suboptimal. The determinants of good compliance to TBP among healthcare providers were making PPE available in the facility, and training healthcare providers on IPC. Tailored public health measures should be implemented to improve and sustain HCPs’ compliance with TBP.

To help reduce the burden of HAIs, which often drive the emergence and spread of AMR, we recommend that policymakers and hospital administrators ensure the supply and availability of PPE to health providers. In addition, functional IPC committees should be established, and HCPs should be encouraged to engage in IPC training emphasizing TBPs.

## Supplementary Information

Additional file 1

Additional file 2

Additional file 3

## Data Availability

All data from the results of this study are available upon reasonable request from the corresponding author.

## Abbreviations

HAI: Healthcare-associated infection
HCP: Healthcare provider
IPC: Infection prevention and control
OBGYN: Obstetrics and gynecology
PPE: Personal Protective Equipment
SP: Standard precautions
TBP: Transmission-based precautions
WHO: World Health Organization
