# Supplementary material for: Compliance with transmission-based precautions, and associated factors among healthcare providers in Cameroon: a cross-sectional study
Source: Antimicrob Resist Infect Control. 2025 Mar 11;14:21. doi: 10.1186/s13756-025-01523-8 (PMC11899890; doi:10.1186/s13756-025-01523-8)
Supplement: Supplementary file 2 — Additional file 2 [file 13756_2025_1523_MOESM2_ESM.docx]

**Compliance Observation form**

| Facility: | Date : |
| --- | --- |
| Ward: | Start time: |
| Shift: morning 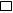 night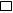 | End time: |
|  | Session duration: |

| Prof.cat | |  | | |
| --- | --- | --- | --- | --- |
| code | |  | | |
| N | |  | | |
| **Opportunity** | **Indication** | | **HH Action** | **Specific indication (s):…………**  **Action** |
| 1 | Before touching patient. 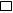  Before-aseptic procedure. 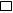  After body fluid exposure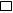 risk.  After touching a patient. 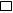  After touching patient 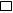surrounding | | **Hand Rub**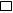  **Hand Washing**  🌕 missed  **🌕 gloves** | **Face Mask**  🌕 missed  **Goggle**  🌕 missed  **Gown** or **apron**  🌕 missed  **Boots**  🌕 missed |
| 2 | Before touching patient. 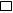  Before-aseptic procedure. 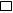  After body fluid exposure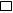 risk.  After touching a patient. 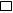  After touching patient 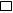surrounding | | **Hand Rub**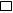  **Hand Washing**  🌕 missed  **🌕 gloves** | **Face Mask**  🌕 missed  **Goggle**  🌕 missed  **Gown** or **apron**  🌕 missed  **Boots**  🌕 missed |
| 3 | Before touching patient. 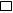  Before-aseptic procedure. 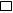  After body fluid exposure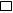 risk.  After touching a patient. 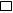  After touching patient 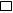surrounding | | **Hand Rub**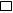  **Hand Washing**  🌕 missed  **🌕 gloves** | **Face Mask**  🌕 missed  **Goggle**  🌕 missed  **Gown** or **apron**  🌕 missed  **Boots**  🌕 missed |

*NB: Glove use may be recorded only when the hand hygiene action is missed while the health-care provider is wearing gloves.*
