# Supplementary material for: Compliance with transmission-based precautions, and associated factors among healthcare providers in Cameroon: a cross-sectional study
Source: Antimicrob Resist Infect Control. 2025 Mar 11;14:21. doi: 10.1186/s13756-025-01523-8 (PMC11899890; doi:10.1186/s13756-025-01523-8)
Supplement: Supplementary file 3 — Additional file 3 [file 13756_2025_1523_MOESM3_ESM.docx]

**Questionnaire on infection prevention and control**

**NB: Complete Sections A, B and C**

**A: General Characteristics**

| S/N | Question | Answers |
| --- | --- | --- |
| 1 | What is your age in years? | /_________________/ yeas |
| 2 | What is your gender? | 1. Male 2. Female |
| 3 | What is your professional rank? | ____________________________________________ |
| 4 | Which department do you work? | ________________________________________ |
| 5 | What is your working shift? | 1. Day 2. Night |
| 6 | What is your years of practice? | 1. <3 years   2. 3-7years  3. >7 years |
| 7 | Have you had Infection Prevention and Control training before? | 1. Yes 2. No |
| 8 | Do you have Infection Prevention and Control committee in your facility? | 1. Yes 2. No |
| 9 | Do you have Personal Protective Equipement available in  your facility? | 1. Yes 2. No |
| 10 | Do you have Infection Prevention and Control guideline in your working department? | 1. Yes 2. No |

**B. Other IPC Characteristics**

| S/N | Questions | Options |
| --- | --- | --- |
| 1 | Have you received the following vaccines? | Hep B vaccin  Covid 19 vaccin |
| 2 | What is your Worker status? | State workers  On contract  Volunteering |
| 3 | What Type is your health facility? | Public  Private |

**C. Knowledge of Infection Prevention and Control**

| **S/N** | **Question** | **Response** |
| --- | --- | --- |
| 1 | Hand washing is necessary before and after procedures are performed. | 1. Yes 2. No |
| 2 | Gloves provide complete protection against  transmission of infections. | 1. Yes 2. No |
| 3 | All needles should be recapped after injection | 1. Yes 2. No |
| 4 | Is the use of an alcohol-based antiseptic for hand hygiene as effective as soap and water if hands are not visibly dirty? | 1. Yes 2. No |
| 5 | Gloves should be worn if blood or body fluid exposure is anticipated | 1. Yes 2. No |
| 6 | Should waste be segregated at the point of generation? | 1. Yes 2. No |
| 7 | Is tuberculosis (TB) carried in airborne particles that are generated from patients with active pulmonary tuberculosis? | 1. Yes 2. No |
| 8 | Is there a need to change gloves between patients as  long as there is no visible contamination? | 1. Yes 2. No |
| 9 | Do you know how to prepare 0.5% chlorine solution? | 1. Yes 2. No |
| 10 | A safety box should be used when three-quarters are full. | 1. Yes 2. No |

NB: A “Yes” in an item gets a score of “1” except for items 2, 3, and 10 where “No” gets a score of "1”. The total score is 10.
